# Supplementary material for: Purine and pyrimidine analogs differentially regulate cell wall precursor biosynthesis to control β-lactam susceptibility in methicillin-resistant Staphylococcus aureus
Source: mBio. 2026 Sep 8;17(10):e01274-26. doi: 10.1128/mbio.01274-26 (PMC13712834; doi:10.1128/mbio.01274-26)
Supplement: Supplementary file 1 [file mbio.01274-26-s0001.docx]

**mBio 01274-26 – REVISED. Purine and pyrimidine analogues differentially regulate cell wall precursor biosynthesis to control β-lactam susceptibility in methicillin resistant *Staphylococcus aureus***

[Aaron C. Nolan](https://orcid.org/0009-0006-1946-0842), [Sarah Byrne](https://orcid.org/0009-0004-9800-5500), [Merve S. Zeden](https://orcid.org/0000-0003-3636-700X)^#^ and [James P. O’Gara](https://orcid.org/0000-0003-3866-7161)^#^

**Supplementary Table and Figures**

**Table S1. Genomic changes in mutants of MRSA strain JE2 with increased resistance to 5-FU, 5-FUrd, 6-TG, Gem and Mito.**

| **Strain** | **Ref. Position** ^a^ | **Type** ^b^ | **Reference** ^c^ | **Allele** ^d^ | **Frequency** ^e^ | **Avg Quality** ^f^ | **Annotations** | **aa change** ^g^ |
| --- | --- | --- | --- | --- | --- | --- | --- | --- |
| 5-FU1 | 484922 | SNV | C | T | 100 | 39.54 | SAUSA300_0432 (Na^+^-dependent transporter) | Thr_12_Ile |
| 5-FU2 | 732454 | SNV | T | C | 100.0 | 39.54 | SAUSA300_0655 (transcriptional regulator) | Ile_219_Thr |
|  | 2527797 | SNV | T | A | 99.89 | 39.54 | SAUSA300_2350 (glutathione S-transferase) | Ile_83_Phe |
| 5-FUrd1 | 131001 | SNV | A | C | 99.7 | 39.66 | Intergenic; upstream of SAUSA300_1994 (*scrB*) | N/A |
|  | 171170 | SNV | T | A | 99.9 | 39.63 | Intergenic; upstream of SAUSA300_0151 (*adhE*) | N/A |
|  | 346185 | SNV | T | G | 100 | 39.83 | Intergenic; upstream of SAUSA300_0292 (hypothetical)) | N/A |
|  | 1957485 | SNV | A | T | 100 | 39.59 | Intergenic; upstream of SAUSA300_1769 | N/A |
|  | 2609133 | SNV | A | C | 100 | 39.82 | Intergenic; upstream of SAUSA300_2423 | N/A |
|  |  | DEL |  | 16 gene DEL |  |  | SAUSA300_0779 - 0815 | N/A |
| 5-FUrd2 | 1493652 | SNV | T | A | 100 | 39.69 | Intergenic; upstream of *ald1* | N/A |
| 5-FUrd3 | 724832 | SNV | T | A | 99.8 | 39.65 | SAUSA300_RS03480 (*pitR*) | Met_202_Lys |
|  | 1190162 | SNV | T | G | 100 | 39.77 | SAUSA300_1088 (glyoxalase) | Val_17_Gly |
|  | 1717440 | SNV | C | T | 100 | 39.86 | SAUSA300_1568 (*udk*) | Asp_135_Asn |
|  | 1840038 | DEL | A | - | 94.5 | 39.41 | Intergenic; upstream of SAUSA300_1679 (*acsA*) | N/A |
|  | 2430693 | SNV | C | T | 99.9 | 39.67 | Intergenic; upstream of SAUSA300_1679 | N/A |
|  | 2430695 | DEL | T | - | 95.5 | 39.31 | Intergenic; upstream of SAUSA300_1679 | N/A |
|  | 2430698 | SNV | T | A | 99.8 | 39.72 | Intergenic; upstream of SAUSA300_1679 | N/A |
|  | 2627112 | DEL | TATA | - | 82.4 | 39.61 | Intergenic; upstream of SAUSA300_2437 (*sarT*) | N/A |
|  |  | DEL |  | 16 gene DEL |  |  | SAUSA300_0779 - 0815 | N/A |
| 6-TG1 | 547123 | SNV | G | A | 100 | 39.72 | SAUSA300_0488 (*hpt*) | Gly_154_Asp |
| 6-TG2 | 547024 | SNV | C | T | 99.9 | 39.76 | SAUSA300_0488 (*hpt*) | Ser_121_Leu |
| Gem1 | 607495 | SNV | C | T | 100 | 39.69 | SAUSA300_0541 (*dck*) | Glu_156_Lys |
|  | 983029 | SNV | A | G | 100 | 39.59 | SAUSA300_0894 (*opp4F*) | Asn_74_Ser |
|  | 1841097 | SNV | G | C | 100 | 39.85 | SAUSA300_1674 (*htrA1*) | Val_337_Leu |
|  |  | DEL |  | 2 gene DEL |  |  | SAUSA300_1822-1823 (tRNA-Met, tRNA-Ser) | N/A |
| Gem2 | 8251 | SNV | C | T | 99.9 | 39.76 | SAUSA300_0006 (*gyrA*) | Ala_407_Val |
|  | 793407 | SNV | C | G | 100 | 39.63 | Intergenic; upstream of SAUSA300_0715 (*nrdI*) | N/A |
|  | 1533985 | SNV | C | A | 100 | 39.49 | SAUSA300_1367 (*cmk*) | Arg_176_Leu |
| Gem3 | 565520 | SNV | C | T | 99.7 | 39.72 | SAUSA300_0506 (*nupC*) | Gly_294_Asp |
| Mito1 | 150826 | SNV | G | C | 100 | 39.88 | SAUSA300_0132 (glycosyltransferase family 1) | Ala_233_Pro |
|  | 591619 | SNV | G | C | 99.5 | 39.76 | SAUSA300_0528 (*rpoC*) | Ala_786_Pro |
|  | 1089834 | SNV | G | A | 99.8 | 39.64 | SAUSA300_0994 (*pdhB*) | Gly_65_Asp |
|  | 1862470 | DEL | T | - | 96.9 | 39.55 | SAUSA300_1687 (*ftsK*) | Asn_316_STOP |
|  | 2148575 | SNV | C | A | 99.8 | 39.83 | SAUSA300_1991 (*agrC*) | Ala_304_Glu |
| Mito2 | 242097 | SNV | G | A | 99.9 | 39.85 | SAUSA300_0204 (*ggt*) | N/A |
|  | 403372 | SNV | G | A | 100 | 39.75 | SAUSA300_0353 (ABC transporter) | Ala_11_Thr |
|  | 591611 | SNV | C | T | 99.8 | 39.29 | SAUSA300_0528 (*rpoC*) | Ala_783_Val |
| Mito3 | 109170 | SNV | C | G | 99.8 | 39.75 | SAUSA300_0099 (*plc*) | N/A |
|  | 428339 | SNV | C | G | 99.8 | 39.63 | SAUSA300_0379 (*ahpF*) | Gly_353_Ala |
|  | 591619 | SNV | G | C | 100 | 39.63 | SAUSA300_0528 (*rpoC*) | Ala_786_Pro |
|  | 709165 | SNV | G | A | 99.9 | 39.68 | SAUSA300_0634 (*fhuB*) | Ala_70_Thr |
|  | 1089957 | SNV | G | A | 100 | 39.79 | SAUSA300_0994 (*pdhB*) | Gly_106_Asp |
|  | 1797730 | SNV | C | G | 99.9 | 39.81 | SAUSA300_1639 (*phoP*) | Met_55_Ile |
|  | 2841479 | SNV | G | A | 99.8 | 39.63 | SAUSA300_2614 (polysaccharide deacetylase) | N/A |

^a^ Reference position: the position in the USA300 FPR3757 genome sequence (NC_007793.1).

^b^ Type of mutation: SNV, single nucleotide variant; DEL, nucleotide deletion.

^c^ Nucleotide base in the USA300 FPR3757 reference genome.

^d^ allele refers to the nucleotide base at the same position in the sequenced strain.

^e^ That is, the percent frequency at which SNV/DEL was found in the sequenced strain.

^f^ Average quality score. A quality score of 30 represents an error rate of 1 in 1,000, with a corresponding call accuracy of 99.9%. N/A, not applicable.

^g^ The amino acid (aa) change denotes the in the protein found in the sequenced strain compared to the reference strain. N/A, not applicable.
